# AI-powered recognition of Chinese medicinal herbs with semantic structure modeling and gradient-guided enhancement

**DOI:** 10.3389/fpls.2025.1672394

**Published:** 2025-11-06

**Authors:** Jiaxing Zou, Ruiwei Liao

**Affiliations:** Guangzhou University of Chinese Medicine, The Research Center of Basic Integrative Medicine, Guangzhou, China

**Keywords:** fine-grained image classification, semantic attention, bidirectional transformer, medicinal plant recognition, deep learning

## Abstract

Digital image processing and object recognition are fundamental tasks in sensor-driven intelligent systems. This paper proposes a structure-aware artificial intelligence framework tailored for fine-grained recognition of medicinal plant images captured by visual sensors. Compared with recent herbal recognition approaches such as CNN enhanced with attention mechanisms, cross-modal fusion strategies, and lightweight transformer variants, our method advances the field by jointly integrating graph-based structural modeling, a Bidirectional Semantic Transformer for multi-scale dependency optimization, and a Gradient Optimization Module for gradient-guided refinement. Built upon a Swin-Transformer backbone, the proposed framework effectively enhances semantic discriminability by capturing both spatial and channel-wise dependencies and adaptively reweighting class-discriminative features. To comprehensively validate the framework, we perform experiments on two datasets: (i) the large-scale TCMP-300 benchmark with 52,089 images across 300 categories, where our model achieves 90.32% accuracy, surpassing the Swin-Base baseline by 1.11%; and (ii) a self-constructed herbal dataset containing 1,872 images across 7 classes. Although the latter is relatively small and not intended as a large-scale benchmark, it serves as a challenging evaluation scenario with high intra-class similarity and complex backgrounds, on which our model achieves 92.75% accuracy, improving by 1.18%. These results demonstrate that the proposed framework not only advances beyond prior herbal recognition models but also provides robust, and sensor-adaptable solutions for practical plant21 based applications.

## 1 Introduction

With the rapid advancement of image recognition technologies, deep learning has achieved notable success in medical diagnostics, crop classification, and Chinese herbal medicine recognition Hao et al. (2021); Liu et al. (2022a). Accurate and efficient herbal image classification is crucial for the digitalization and intelligent transformation of traditional Chinese medicine (TCM), supporting traceability, quality control, education, and clinical applications. However, the diversity, complex morphology, and varying imaging conditions of herbal samples present significant challenges, leading to limited generalization and weak discriminative power in real-world scenarios. Developing a framework with robust feature extraction and strong structural perception is therefore of high research value Min and Yingchong (2025).

Although convolutional neural networks (CNNs) and visual Transformers have been widely used in image recognition, they remain insufficient for fine-grained herbal classification Sharma et al. (2023). First, conventional backbones focus on local textures and struggle to capture complex global structures. Second, many models cannot effectively integrate multi-scale features, reducing performance when recognizing organs or rhizomes of varying scales Borman et al. (2021). Third, feature learning is often performed without explicit optimization guidance, resulting in low inter-class separability and unstable performance Chaivivatrakul et al. (2022).

To address these issues, we propose a structural-aware herbal image recognition framework with contextual structural modeling and gradient-based optimization. The model includes three components: (1) a Swin-Transformer encoder for hierarchical and global visual features; (2) a Bidirectional Transformer to enhance multi-scale semantic structures and contextual dependencies; and (3) a Gradient Optimization Module with channel-wise attention and backpropagation-guided refinement for stronger discriminative learning. Experiments on two herbal datasets demonstrate the superior accuracy, robustness, and generalization of our approach.

The key contributions of this work are summarized as follows:

- We propose a Bidirectional Transformer-based structural enhancement module to effectively model the multi-scale relationships within herbal images, improving semantic representation of complex categories.
- We design a novel Gradient Optimization Module that leverages backpropagation gradients to guide channel attention and refine feature learning, enhancing inter-class discrimination.
- We construct a custom herbal dataset covering seven representative categories and conduct comprehensive evaluations on this dataset and TCMP-300, demonstrating the robustness and interpretability of our framework.

## 2 Related work

### 2.1 Image recognition algorithms

In recent years, image recognition technologies have been widely applied in various domains such as medical diagnosis, smart agriculture, autonomous driving, and industrial inspection. The core driving force behind this trend lies in the breakthroughs of deep learning models in feature extraction and pattern recognition. Convolutional Neural Networks (CNNs) remain the dominant framework for image recognition tasks. Liu et al. (2024) reviewed the latest advances in lightweight CNN architectures, emphasizing the potential of models such as ShuffleNet and MobileNet for deployment in embedded systems. Han et al. (2024) further proposed a latency-aware dynamic network architecture, which successfully reduces inference delay while maintaining recognition accuracy, thereby improving deployment efficiency in edge computing scenarios. Addressing the challenge of multimodal feature fusion, Wang et al. (2024) introduced a deep multimodal fusion framework that significantly enhances robustness in complex visual environments.

At the same time, emerging methods continue to diversify and advance image recognition algorithms. Yadav et al. (2024) developed a smart image recognition model that integrates fuzzy logic with deep neural networks, enhancing decision-making under uncertain conditions. Galic´ et al. (2024) surveyed representative applications of neural networks and machine learning in image recognition, highlighting future trends such as model compression, automatic feature selection, and cross-modal representation learning. Building on this, HASAN et al. (2024) experimentally demonstrated the generalization and robustness of deep convolutional networks for multi-class image classification, particularly in the context of data augmentation and hierarchical feature fusion. Furthermore, Koziolek and Koziolek (2024) proposed a cross-modal code generation framework that integrates image recognition with large language models (LLMs), showcasing the potential of visual semantic understanding in intelligent programming.

It is worth noting that task-specific image recognition algorithms continue to evolve. Basha et al. (2025) introduced a quantum dilated CNN architecture that combines quantum computation with image recognition, achieving efficient pattern recognition and feature compression. Xiang et al. (2024) proposed a highly robust algorithm for spliced image detection based on image statistical characteristics, significantly improving the accuracy of forgery detection. In summary, current image recognition algorithms are shifting from single-stream convolutional modeling toward multidimensional integration across modalities, domains, and resource-constrained environments. With continued innovations in model architecture and computational efficiency, image recognition is expected to expand its practical boundaries under the dual demand for high accuracy and low cost.

### 2.2 Applications of image recognition in Chinese herbal medicine

In recent years, the application of image recognition technology to Chinese herbal medicine identification has achieved significant progress, with deep learning emerging as the core technical path for improving classification accuracy. Shang and Wang (2024) proposed an intelligent recognition framework based on deep neural networks, which effectively enhanced robustness under complex backgrounds. Hou et al. (2025) further introduced knowledge distillation and cross-attention mechanisms to design a lightweight model tailored for Chinese herb image recognition, achieving not only improved accuracy but also reduced inference cost. Chen et al. (2021) developed an automated recognition approach based on the AutoML platform, capable of identifying over 300 commonly used medicinal herbs with high practical utility.

From the methodological perspective, research in this field has shown increasing diversity in recent years. Ye et al. (2024) employed deep convolutional networks to extract multi-scale features for multi-class classification of herbal images, enabling recognition across texture and structural levels. Focusing on microscopic image features, Pang et al. (2022) proposed an automatic identification method that incorporates microscopic processing, achieving excellent performance in distinguishing morphologically similar species. Pan et al. (2024) combined AI-based machine learning with deep learning strategies to systematically explore the potential of image recognition in multi-task scenarios within traditional Chinese medicine, including efficacy evaluation, sample screening, and prescription modeling. Sha et al. (2025) introduced the HerbMet framework, integrating image recognition with metabolomics data to support precise identification and component analysis of complex medicinal materials.

Collectively, these studies demonstrate that image recognition technology not only addresses the limitations of expert-dependent traditional identification but also significantly improves the efficiency and consistency of recognition. A scientometric analysis by Tian et al. (2023) revealed that image recognition has become one of the key research directions in AI-driven traditional Chinese medicine, particularly in the digital transformation of visual diagnosis. Mulugeta et al. (2024) conducted a systematic review of deep learning methods for medicinal plant classification, emphasizing future research trends in cross-modal fusion, few-shot learning, and enhanced interpretability. Additionally, Meng et al. (2023) highlighted the auxiliary role of remote sensing image recognition in 116 estimating cultivation areas and analyzing species distribution of Chinese medicinal herbs. Overall, the integration of knowledge graphs, image recognition, and multimodal sensing is expected to be a critical pathway toward the practical and intelligent development of Chinese herbal image recognition.

## 3 Methodology

### 3.1 Overall model architecture

**Figure 1:**
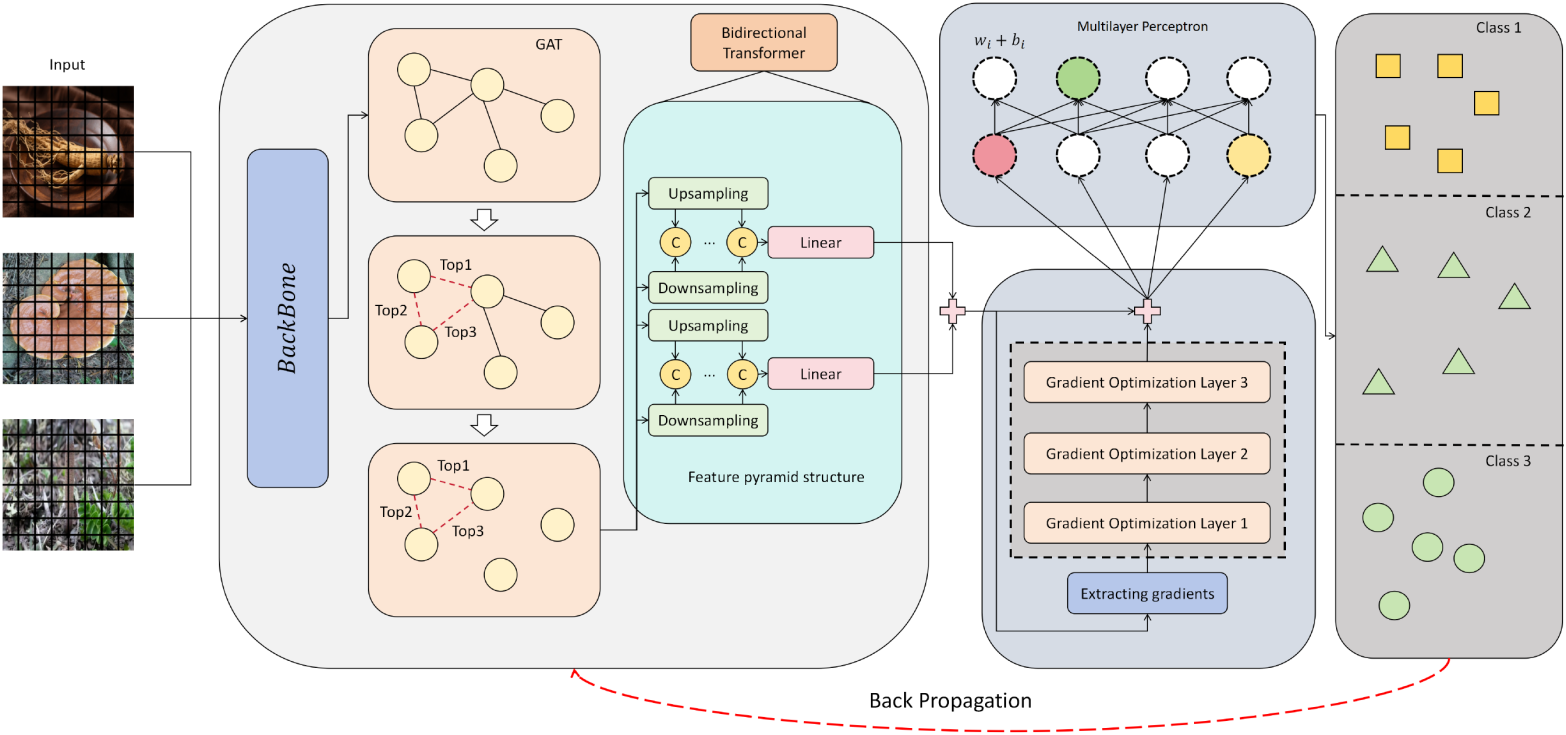
Overall architecture of the proposed herbal image recognition model. The model employs a SwinTransformer backbone augmented with a Graph Attention Network to capture relational dependencies, incorporates a feature pyramid with a bidirectional transformer for multi-scale semantic fusion, and integrates a gradient optimization module for enhanced representation learning.

This study proposes a novel herbal image recognition framework that integrates graph neural structure perception and a bidirectional transformer optimization mechanism. As illustrated in **Figure 1**, the model takes herb image samples as input, first extracting initial semantic features through a visual backbone. It then constructs topological relations among regions via a simple Graph Attention Network (GAT) module, which serves as the core mechanism for capturing region-level structural dependencies rather than an ablation-only variant. Specifically, each feature position is treated as a graph node and aggregated through attention-based message passing, thereby enhancing the structural representation of herbal organs. The graph-aware features are subsequently fed into a bidirectional transformer for contextual interaction and reconstruction, yielding a set of discriminative embeddings for classification. By jointly leveraging local spatial structure and global semantic context, the architecture aims to enhance fine-grained recognition under complex texture backgrounds. Compared with recent CNN+attention models that primarily focus on local discriminative regions, our method explicitly incorporates graph-based structural modeling to capture relational dependencies across herbal organs. In contrast to cross-modal fusion approaches, our design emphasizes purely visual cues while introducing gradient-guided optimization for enhanced interpretability. Furthermore, unlike lightweight transformers that prioritize efficiency, the proposed framework achieves a balanced improvement in both accuracy and robustness through the synergistic integration of graph-aware structural modeling, bidirectional contextual modeling, and gradient-aware channel recalibration.

Specifically, given an input image $X\in {\mathbb{R}}^{H\times W\times 3}$, a hierarchical visual backbone—namely, the Swin Transformer—is employed to generate multi-scale feature maps ${\left\{F_{l}\right\}}_{l=1}^{L}$, where each $F_{l}\in {\mathbb{R}}^{H_{l}\times W_{l}\times C_{l}}$ represents a feature embedding at a specific resolution. The Swin Transformer partitions the image into non overlapping windows and applies window-based self-attention. Shifted window operations are introduced across layers to enable cross-region modeling. The computations are formally defined as:

(1) $$Z_{l}=\text{W}-\text{MSA}(\text{LN}(F_{l-1}))+F_{l-1},$$

(2) $$F_{l}=\text{MLP}(\text{LN}(Z_{l}))+Z_{l},$$

where W-MSA denotes the Window-based Multi-head Self-Attention module, LN is Layer Normalization, and MLP represents a feed-forward network.

On top of the backbone features, we design a graph-aware representation mechanism by incorporating a Graph Attention Network (GAT) to enhance region-level structural expression. Each position in the feature map **F***_l_* is treated as a graph node. An adjacency graph G=(V,E) is constructed based on Euclidean distances or semantic similarity, and node features are aggregated using attention. The update rule for each node is given by:

(3) $$h_{i}^{\prime }=\sigma \left(\sum_{j\in N\left(i\right)}\alpha_{ij}Wh_{j}\right)\;,$$

where *α_ij_* denotes the attention coefficient between node *i* and *j*, **W** is a learnable linear projection, *σ*(·) is an activation function, and N(*i*) represents the neighborhood of node *i*. To improve the robustness of graph representation, multiple layers of GAT are stacked to capture higher-order semantic connectivity.

Based on the graph-aware features, a feature pyramid structure is introduced to adaptively fuse multi-scale graph embeddings, including a top-down upsampling path and a bottom-up downsampling path. At each level, graph features are concatenated and linearly projected to a unified dimension before being fed into a bidirectional transformer to capture inter-layer semantic consistency and cross-scale contextual dependencies. The fusion process is formulated as:

(4) $$f_{\text{fuse}}^{l}=\text{Linear}([\text{Up}(h^{l+1});h^{l};\text{Down}(h^{l-1})]),$$

where Up(·) and Down(·) denote upsampling and downsampling operations respectively, [·;·] is concatenation, and Linear(·) indicates a linear transformation.

Finally, the fused feature sequence is input to the Bidirectional Transformer for long-range dependency modeling and feature interaction, which provides a globally consistent embedding foundation for subsequent optimization and classification.

### 3.2 Bidirectional self-attention-based transformer architecture

**Figure 2:**
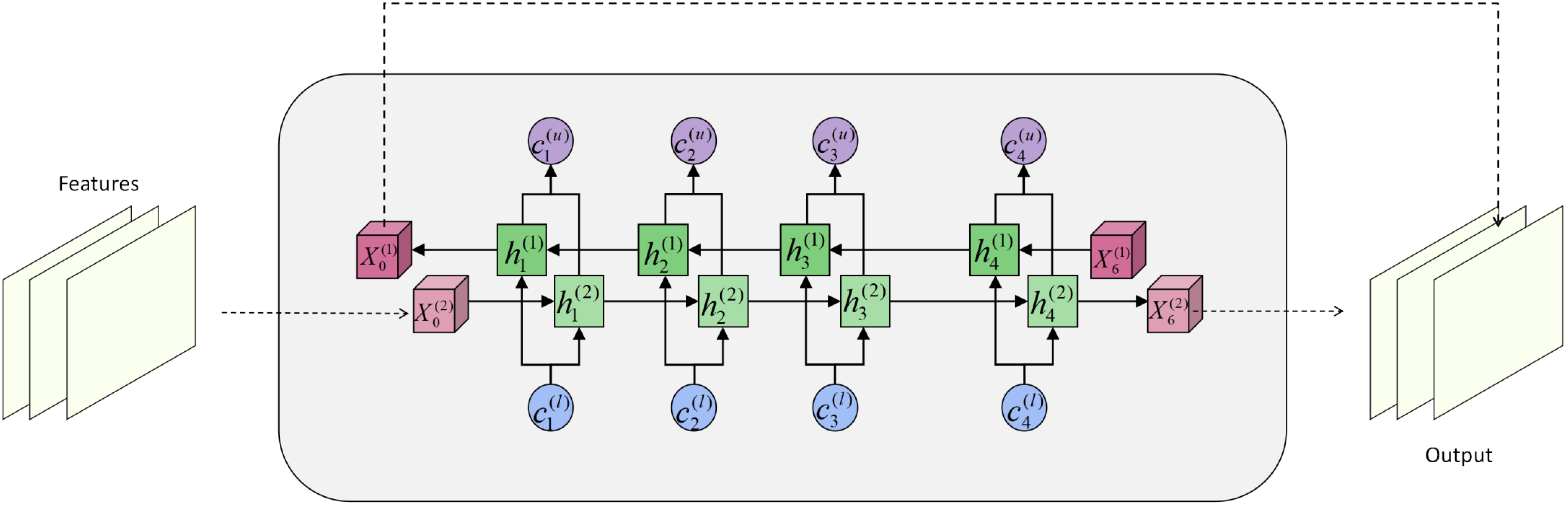
Diagram of the bidirectional transformer architecture, illustrating the information interaction and state update mechanisms between the forward and backward paths.

To further enhance long-range dependency modeling of fine-grained semantics in herbal images, we introduce a Bidirectional Transformer structure during the feature fusion stage, enabling global context modeling across both channels and temporal steps. Inspired by bidirectional sequence modeling, this module is designed to capture both forward and backward dependencies simultaneously, thereby strengthening the coupling between spatial structure and semantic representation. Its module architecture is shown in **Figure 2**.

Given the fused sequence features ${\left\{X_{t}\right\}}_{t=1}^{T}$ from the graph attention and feature pyramid modules, where each $X_{t}\in {\mathbb{R}}^{C\times H\times W}$ denotes the feature at time step t, we first flatten the spatial dimensions to obtain a 2D representation suitable for Transformer input: ${\tilde{X}}_{t}=\text{Flatten}(X_{t})\in {\mathbb{R}}^{N\times d}$, where N=H·W is the number of tokens and d is the embedding dimension.

Within each encoder layer, we construct bidirectional attention paths comprising forward and backward self-attention. Given the input ${\tilde{X}}_{t}^{\left(l\right)}$ at the l-th layer, the forward attention output is computed as:

(5) $$Z_{t}^{\left(f\right)}=\text{Softmax}(\frac{Q_{t}^{\left(f\right)}{\left(K_{t}^{\left(f\right)}\right)}^{⊤}}{\sqrt{d}})V_{t}^{\left(f\right)},$$

where $Q_{t}^{\left(f\right)}={\tilde{X}}_{t}^{\left(l\right)}W_{Q}^{\left(f\right)}$, $K_{t}^{\left(f\right)}={\tilde{X}}_{t}^{\left(l\right)}W_{K}^{\left(f\right)}$, and $V_{t}^{\left(f\right)}={\tilde{X}}_{t}^{\left(l\right)}W_{V}^{\left(f\right)}$ represent the query, key, and value matrices in the forward path. The backward path propagates from the end of the sequence and is defined as:

(6) $$Z_{t}^{\left(b\right)}=\text{Softmax}(\frac{Q_{t}^{\left(b\right)}{\left(K_{t}^{\left(b\right)}\right)}^{⊤}}{\sqrt{d}})V_{t}^{\left(b\right)},$$

where $Q_{t}^{\left(b\right)}={\tilde{X}}_{T-t+1}^{\left(l\right)}W_{Q}^{\left(b\right)}$, with the other definitions consistent. The two attention outputs are then fused to obtain a context-enhanced representation:

(7) $$Z_{t}=\gamma \cdot Z_{t}^{\left(f\right)}+(1-\gamma )\cdot Z_{t}^{\left(b\right)},$$

where *γ* ∈ [0,1] is a learnable fusion coefficient that dynamically adjusts the weighting of forward and backward information.

Each Transformer block contains a feed-forward sub-layer for temporal modeling. To further enhance channel-wise interactions, we integrate a channel attention mechanism that adaptively reweights the context representation **Z***_t_*. For the *c*-th channel feature vector **z***_c_*∈ ℝ*^N^*, the attention activation is defined as:

(8) $$\alpha_{c}=\sigma (W_{2}\cdot \delta (W_{1}\cdot \text{GAP}(z_{c}))),$$

where GAP(·) denotes global average pooling, *δ*(·) and *σ*(·) denote the ReLU and Sigmoid functions, and *W*_1_, *W*_2_ are learnable weight matrices. The recalibrated output is then given by:

(9) $${\hat{z}}_{c}=\alpha_{c}\cdot z_{c}.$$

Structurally, the Bidirectional Transformer is implemented in a stacked manner to facilitate deep context fusion, with each layer employing residual connections and layer normalization to ensure stable gradient propagation. Let *F_l_*(·) denote the *l*-th Transformer operation, the output at the (*l* + 1)-th layer is formulated as:

(10) $${\tilde{X}}_{t}^{\left(l+1\right)}=\text{LayerNorm}({\tilde{X}}_{t}^{\left(l\right)}+F_{l}({\tilde{X}}_{t}^{\left(l\right)})).$$

To further capture inter-temporal interactions, we adopt a cross-frame concatenation mechanism by sequentially stacking features from different time steps and projecting them into the classification space. The temporally stacked representation is defined as:

(11) $$H=\text{Concat}({\tilde{X}}_{1}^{\left(L\right)},{\tilde{X}}_{2}^{\left(L\right)},\ldots ,{\tilde{X}}_{T}^{\left(L\right)})\cdot W_{p},$$

where $W_{p}\in {\mathbb{R}}^{dT\times d^{\prime }}$ is a learnable projection matrix, and *d*^′^ denotes the target embedding dimension for downstream tasks. The final output **H** is passed to the embedding optimization module for semantic compression and classification.

In summary, the Bidirectional Transformer module plays a pivotal role in our framework by introducing bidirectional information interaction, channel attention modulation, and cross-temporal feature aggregation. This effectively compensates for the limitations of convolutional architectures in long-range modeling and semantic consistency, providing strong structural support for capturing complex texture relationships in herbal images.

### 3.3 Gradient optimization module

**Figure 3:**
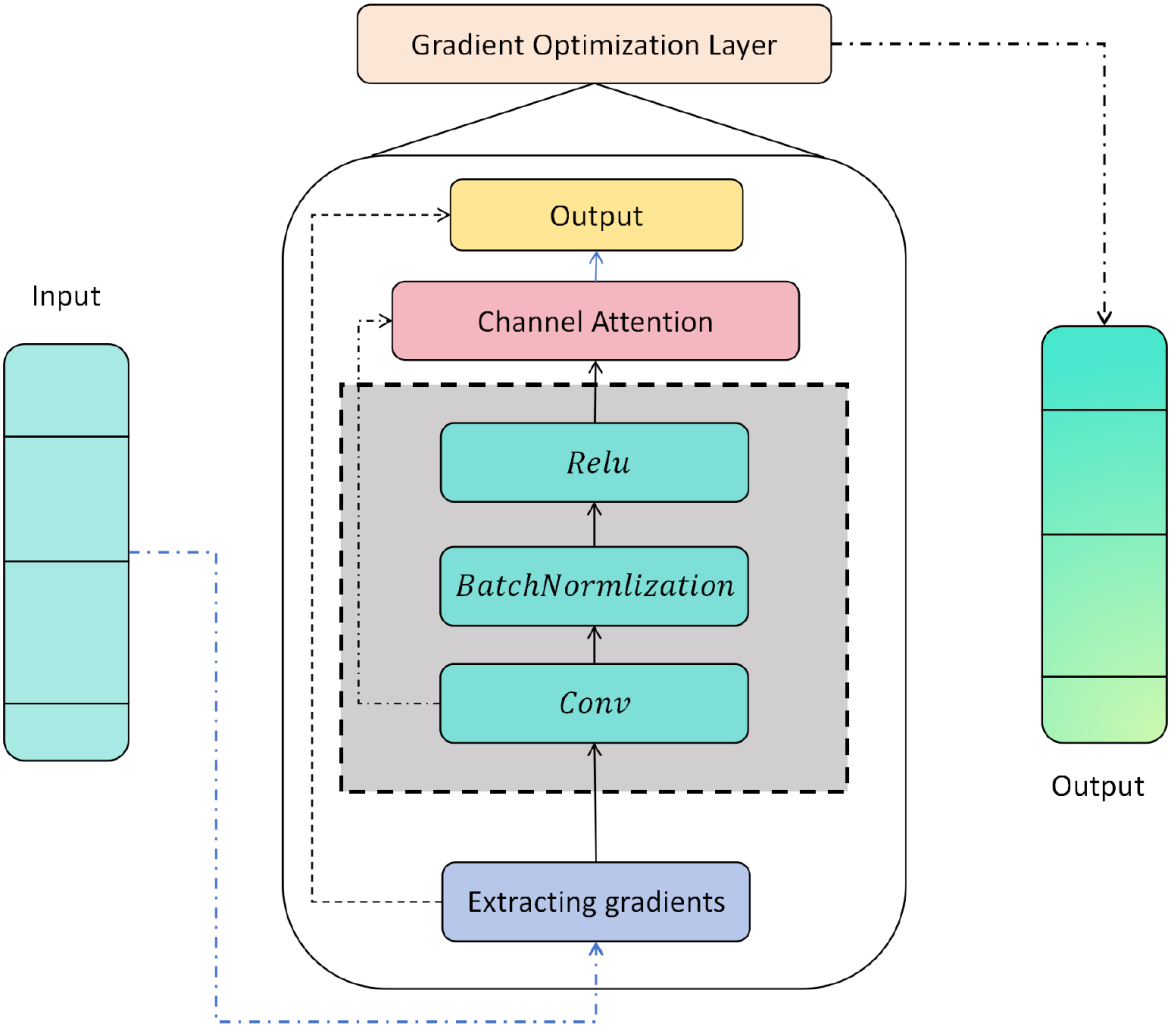
Schematic diagram of the gradient optimization module, showing the feature recalibration process achieved by combining gradient extraction, convolutional reconstruction, and channel attention.

After obtaining the globally consistent representation **H** ∈ R*^C^*^×^*^H^*^×^*^W^* from the Bidirectional Transformer, we further introduce a Gradient Optimization Module (GOM) to identify the most discriminative channels and spatial positions within the feature map. The core idea of this module is to explicitly extract backward 206 gradient signals during forward propagation and use them as saliency guidance for adaptively reweighting 207 convolutional channels, thereby achieving gradient-aware feature enhancement. To minimize redundant 208 computation, gradient extraction is performed only once on the output **H** and reused within the same 209 memory context as needed. Its module architecture is shown in **Figure 3**.

Let J denote the overall network objective. The gradient with respect to the feature map is defined as:

(12) $$G=\frac{\partial J}{\partial H}\in {\mathbb{R}}^{C\times H\times W}.$$

To alleviate instability caused by gradient magnitude variance, we apply *L*_2_ normalization:

(13) $$\hat{G}=\frac{G}{∥G{∥}_{2}+\epsilon },$$

where *ϵ* is a small constant added to prevent division by zero. We then perform global average pooling over each channel *c* to compute the gradient intensity vector:

(14) $$g_{c}=\frac{1}{HW}\sum_{i=1}^{H}\sum_{j=1}^{W}|{\hat{G}}_{c,i,j}|.$$

A learnable linear projection is applied to derive the gating coefficient:

(15) $$\beta_{c}=\sigma (W_{g}\;g_{c}+b_{g}),$$

where *W_g_*and *b_g_*are the weight and bias parameters, and *σ*(·) denotes the Sigmoid activation function. The coefficient *β_c_*∈ (0,1) reflects the importance of channel *c* for the final classification task.

Guided by these gradient-based weights, the original feature map **H** is fed into a local reconstruction path consisting of convolution, batch normalization, and nonlinear activation. Let **W**_conv_ denote the convolutional kernel, then the output of the convolutional layer is:

(16) $$U=W_{\text{conv}}*H,$$

followed by batch normalization:

(17) V=BN(U),

and a nonlinear activation function ϕ(·) (ReLU in this work):

(18) Z=ϕ(V).

Channel-wise recalibration is then applied via element-wise multiplication to form the gradient-enhanced representation:

(19) $${\tilde{Z}}_{c,:,:}=\beta_{c}⊙Z_{c,:,:}.$$

To maintain feature distribution stability and promote progressive integration of deep features, the module incorporates a residual connection that adds the enhanced representation to the original input:

(20) $$H^{\prime }=\tilde{Z}+H.$$

This output not only preserves the long-range dependencies captured by the Bidirectional Transformer but also emphasizes the most class-discriminative channel responses through gradient-based modulation, thereby improving recognition accuracy without introducing additional loss constraints. The Gradient Optimization Module is lightweight in parameter overhead and computational cost, and can be seamlessly integrated with stacked Transformer layers to provide interpretable and dynamic feature enhancement.

### 3.4 Training objective

To fully exploit the long-range dependency and channel saliency information captured by the Bidirectional Transformer and the Gradient Optimization Module, we formulate the overall training objective as a combination of classification cross-entropy, gradient consistency regularization, and weight decay terms.

Given the predicted class probability $\hat{y}$*_n_*∈ ℝ*^K^* from the classification head and the one-hot ground truth label **y***_n_*, the standard cross-entropy loss is defined as:

(21) $$L_{\text{CE}}=-\frac{1}{N}\sum_{n=1}^{N}y_{n}^{⊤}\text{log }{\hat{y}}_{n},$$

which encourages the predicted distribution to align closely with the true labels.

To enforce semantic consistency between the channel weights *β* learned in the Gradient Optimization.

Module and the normalized gradient intensities **g**˜, we introduce a gradient consistency regularization term:

(22) $$L_{\text{G}}=\frac{1}{C}\sum_{c=1}^{C}∥\beta_{c}-{\tilde{g}}_{c}{∥}_{2}^{2},$$

which serves to stabilize the learning of salient channels throughout training.

In parallel, we apply *L*_2_ regularization to all learnable parameters Θ to mitigate overfitting:

(23) $$L_{\text{WD}}=∥\text{Θ}{∥}_{2}^{2}.$$

The final loss function integrates all components as:

(24) $$L=L_{\text{CE}}+\lambda_{1}L_{\text{G}}+\lambda_{2}L_{\text{WD}},$$

where *λ*_1_ and *λ*_2_ are hyperparameters that can be tuned on a validation set to achieve an optimal balance between discriminative capability and generalization performance. In our experiments, we empirically set *λ*_1_ = 0.5 and *λ*_2_ = 0.5 to ensure stable training and balanced optimization.

## 4 Dataset and experimental settings

### 4.1 Dataset

#### 4.1.1 TCMP-300

**Figure 4:**
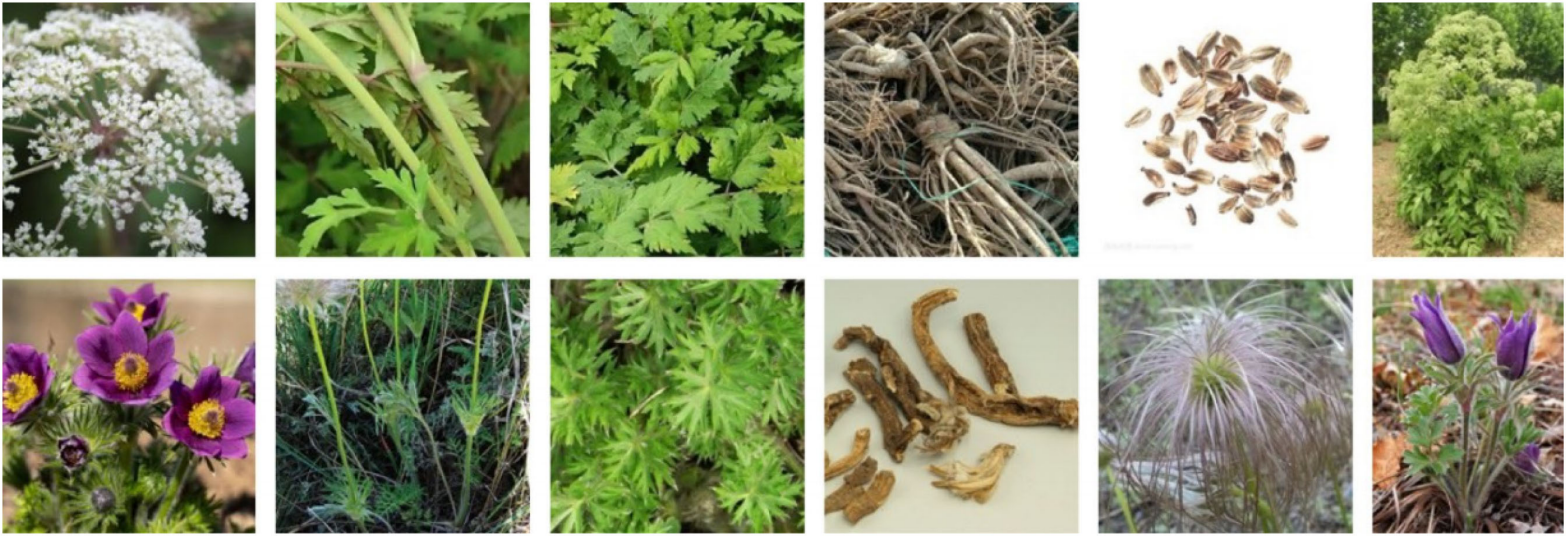
TCMP-300 dataset example Zhang et al. (2025).

The TCMP-300 Zhang et al. (2025) dataset is one of the most comprehensive publicly available benchmarks for Chinese herbal image recognition, comprising a total of 52,089 images across 300 distinct categories of traditional Chinese medicinal materials. It significantly surpasses previous datasets in both scale and diversity. Images were automatically crawled from the Bing search engine using scripted pipelines, then preliminarily filtered with vision foundation models such as CLIP and manually verified by traditional Chinese medicine (TCM) experts to ensure high quality and annotation accuracy. The dataset covers six representative plant structures—flowers, leaves, stems, fruits, roots, and whole herbs—demonstrating rich fine-grained details and structural variety. Moreover, TCMP-300 incorporates a HybridMix data augmentation strategy and provides benchmark evaluations using state-of-the-art models such as the Swin Transformer, validating its robustness and practicality under multi-view and complex background conditions. This dataset establishes a reliable foundation and evaluation protocol for future research in automated Chinese herbal identification. An example of the dataset is shown in **Figure 4**.

### 4.2 Self-constructed dataset

**Figure 5:**
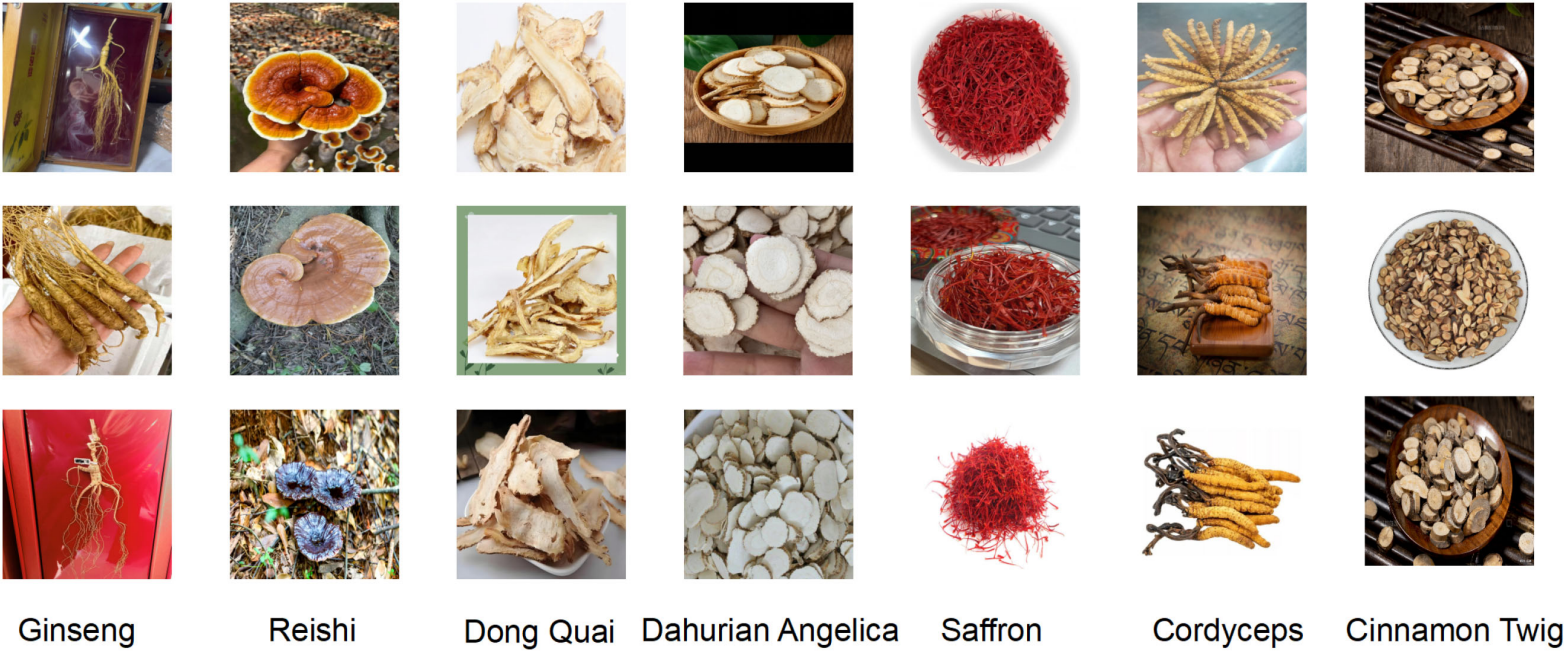
This article’s self-made dataset example.

To enhance recognition accuracy for specific herbal categories, we additionally constructed a custom dataset containing 1,872 images across 7 representative classes of commonly used Chinese medicinal herbs, including *Panax ginseng* (268 images), *Ganoderma lucidum* (270 images), *Angelica dahurica* (265 images), *Cinnamomi ramulus* (267 images), *Crocus sativus* (269 images), *Cordyceps sinensis* (266 images), and *Angelica sinensis* (267 images). All images were standardized to a resolution of 640 × 640 pixels to ensure consistency in training and evaluation. The dataset was constructed through a combination of web scraping from open-access online repositories, retrieval from herbarium databases, and field photography conducted by researchers. Each image was screened and validated independently by two experts in traditional Chinese medicine, and disagreements were resolved through discussion to ensure annotation consistency. The images reflect real-world conditions under varying angles, lighting, and background settings. Designed as a structurally simple and semantically clear medium-scale benchmark, this dataset complements large-scale public datasets by addressing the limited coverage of certain herbal categories and evaluating the model’s generalization and adaptability in fine-grained recognition tasks. An example of this dataset is shown in **Figure 5**.

### 4.3 Experimental settings

In our image classification experiments, we adopt the Swin-Transformer Base as the visual backbone, with model parameters initialized from publicly available ImageNet-pretrained weights to enhance feature generalization and accelerate convergence. Prior to training, all herbal images are resized to 224 × 224 pixels, normalized using the ImageNet mean and standard deviation, and color normalization to reduce the influence of illumination variations. To further improve model robustness, we apply a series of augmentation strategies, including random cropping, horizontal flipping, rotation within ±15°, color jittering, and random erasing, which simulate real-world variability in imaging conditions and morphological perspectives. The data augmentation table is shown in **Table 1**.

**Table 1:** Data augmentation strategies used in herbal image classification experiments.

Augmentation	Description
Resize	All images resized to 224 × 224 pixels
Normalization	ImageNet mean and standard deviation
Color normalization	Adjust color distribution to reduce illumination effects
Random cropping	Crop random regions within the image
Horizontal flipping	Flip images horizontally with probability 0.5
Rotation	Random rotation within ±15°
Color jittering	Random changes in brightness, contrast, saturation, and hue
Random erasing	Randomly erase rectangular regions in the image

All models are trained and evaluated on both the TCMP-300 dataset and the self-constructed herbal dataset. The training process spans 200 epochs, utilizing the AdamW optimizer with a cosine annealing learning rate schedule. A grid search strategy is employed to tune key hyperparameters such as learning rate, weight decay, and batch size, ensuring fair and optimized comparisons across different baselines. During both training and evaluation, we employ four standard metrics—Accuracy, Precision, Recall, and F1-score—to comprehensively assess the model’s performance on fine-grained herbal recognition tasks. The detailed experimental settings are summarized in **Table 2**.

**Table 2:** Configuration of experimental settings.

Setting	Value
Backbone Network	Swin-Transformer Base
Pretrained Weights Source	ImageNet
Training Epochs	200
Optimizer	AdamW
Learning Rate Schedule	Cosine Annealing
Batch Size	64
Input Resolution	224 × 224
Evaluation Metrics	Accuracy, Precision, Recall, F1-score
Data Augmentation	Standard random crop and flip

## 5 Experimental results

### 5.1 Comparative experimental results

To comprehensively evaluate the performance advantages of the proposed model in Chinese herbal image recognition, we conduct extensive comparative experiments against several mainstream architectures, including both convolutional neural networks and vision transformers. The selected baseline models consist of ResNet50 He et al. (2016), VGG19 Simonyan and Zisserman (2014), ResNeXt Xie et al. (2017), ConvNeXt Liu et al. (2022b), ViT Dosovitskiy et al. (2020), and Swin-Transformer Liu et al. (2021). Under identical datasets and training protocols, we systematically compare their performance in terms of classification accuracy, precision, recall, and F1-score. This evaluation is intended to validate the effectiveness of our architecture in capturing fine-grained semantic features and modeling long-range dependencies. All models are initialized with ImageNet-pretrained weights and trained under the same number of epochs and input specifications, ensuring fairness, reproducibility, and reliability of the experimental results. This paper first gives the experimental results of TCMP, as shown in **Table 3**.

**Table 3:** Comparison of classification performance across different models on the TCMP-300 dataset with computational efficiency (mean ± std over three independent runs).

Model	Params (M)	Inference (ms)	Accuracy (%)	Precision (%)	Recall (%)	F1-score (%)
VGG19	143.7	12.6	86.32 ± 0.18	85.74 ± 0.21	84.98 ± 0.25	85.36 ± 0.20
ResNet50	25.6	8.7	86.51 ± 0.22	85.93 ± 0.24	85.22 ± 0.27	85.57 ± 0.23
ResNeXt	24.7	9.1	88.21 ± 0.20	87.80 ± 0.19	87.02 ± 0.23	87.41 ± 0.21
ConvNeXt	28.6	9.8	88.37 ± 0.17	87.96 ± 0.20	87.30 ± 0.22	87.63 ± 0.19
ViT	86.1	13.4	83.25 ± 0.25	82.68 ± 0.23	81.92 ± 0.28	82.30 ± 0.26
Swin-Tiny	28.3	7.9	87.32 ± 0.21	86.84 ± 0.22	86.12 ± 0.24	86.48 ± 0.22
Swin-Small	51.3	9.3	86.96 ± 0.19	86.42 ± 0.20	85.79 ± 0.22	86.10 ± 0.21
Swin-Base	88.0	12.7	89.21 ± 0.16	88.92 ± 0.18	88.15 ± 0.19	88.53 ± 0.17
Ours	92.1	12.9	90.32 ± 0.14	90.01 ± 0.15	89.43 ± 0.16	89.72 ± 0.15

The classification performance comparison on the TCMP-300 dataset demonstrates that the proposed model consistently outperforms existing mainstream methods across multiple evaluation metrics, highlighting its superiority in Chinese herbal image recognition. Specifically, while the Swin-Transformer Base already exhibits strong capabilities in modeling long-range dependencies with an accuracy of 89.21%, it is still marginally surpassed by our model, which achieves 90.32% accuracy. This improvement is attributed to the integration of the Bidirectional Transformer and the Gradient Optimization Module on top of the base architecture. The former significantly enhances global information exchange across multi scale contexts, while the latter emphasizes discriminative semantic responses among channels through gradient-guided recalibration.

Compared to traditional convolutional networks such as ResNet50 and VGG19, our model achieves approximately 4% and 4.3% improvements in accuracy and F1-score, respectively. This indicates that models relying solely on local receptive fields are limited when handling the complex structures and fine textures inherent in herbal images. Moreover, in comparison with enhanced CNN variants like ResNeXt and ConvNeXt, our method still delivers over 2% performance gains, further validating the effectiveness of the graph-aware and bidirectional modeling mechanisms in improving recognition accuracy. Notably, the ViT model exhibits relatively weaker performance on this task, likely due to its lack of local structure modeling, which hinders its ability to extract stable key features in datasets characterized by substantial morphological variability.

Overall, the proposed architecture maintains a balanced trade-off between computational efficiency and representational expressiveness, achieving both semantic consistency and structural sensitivity. This makes it particularly suitable for herbal image classification under diverse categories, morphological variations, and non-standard imaging conditions. The results not only confirm the method’s effectiveness on large-scale public datasets but also offer a robust technical foundation and methodological paradigm for intelligent Chinese herbal image recognition.

Furthermore, we also report the results on the custom dataset, with the experimental outcomes summarized in **Table 4**.

**Table 4:** Comparison of classification performance across different models on the self-built herbal dataset with computational efficiency (mean ± std over three independent runs).

Model	Params (M)	Inference (ms)	Accuracy (%)	Precision (%)	Recall (%)	F1-score (%)
VGG19	143.7	12.6	89.14 ± 0.20	88.73 ± 0.21	88.20 ± 0.23	88.46 ± 0.22
ResNet50	25.6	8.7	90.37 ± 0.18	90.02 ± 0.19	89.74 ± 0.21	89.88 ± 0.20
ResNeXt	24.7	9.1	91.08 ± 0.17	90.87 ± 0.18	90.41 ± 0.20	90.64 ± 0.19
ConvNeXt	28.6	9.8	91.33 ± 0.15	91.05 ± 0.17	90.58 ± 0.18	90.81 ± 0.17
ViT	86.1	13.4	90.21 ± 0.22	89.97 ± 0.23	89.52 ± 0.25	89.74 ± 0.24
Swin-Tiny	28.3	7.9	90.96 ± 0.16	90.61 ± 0.18	90.19 ± 0.19	90.40 ± 0.18
Swin-Small	51.3	9.3	90.72 ± 0.17	90.38 ± 0.18	89.91 ± 0.20	90.14 ± 0.19
Swin-Base	88.0	12.7	91.57 ± 0.14	91.26 ± 0.16	90.83 ± 0.17	91.04 ± 0.15
Ours	92.1	13.2	92.75 ± 0.12	92.41 ± 0.13	91.94 ± 0.14	92.17 ± 0.13

The experimental results on the custom Chinese herbal image dataset further validate the generalization ability and classification stability of the proposed model under low-data and low-class-count conditions. Although the dataset comprises only seven representative herb categories, its diverse imaging conditions and complex backgrounds present significant challenges for fine-grained recognition. As observed from the comparison, conventional CNN models such as VGG19 and ResNet50 demonstrate relatively stable performance but remain limited in capturing subtle texture variations and structural differences inherent in herbal images. Transformer-based models, including the Swin series and ViT, achieve higher accuracy through global modeling capabilities, yet exhibit certain limitations in feature localization and channel discrimination. In contrast, the synergistic integration of the Bidirectional Transformer and the Gradient Optimization Module in our method effectively enhances the coupling between local textures and global structures, enabling robust and semantically consistent recognition across varying lighting and pose conditions. Ultimately, the proposed model achieves the best performance across all evaluation metrics, demonstrating superior adaptability and expressive power in fine-grained classification tasks characterized by high intra-class similarity and morphological diversity.

**Figure 6:**
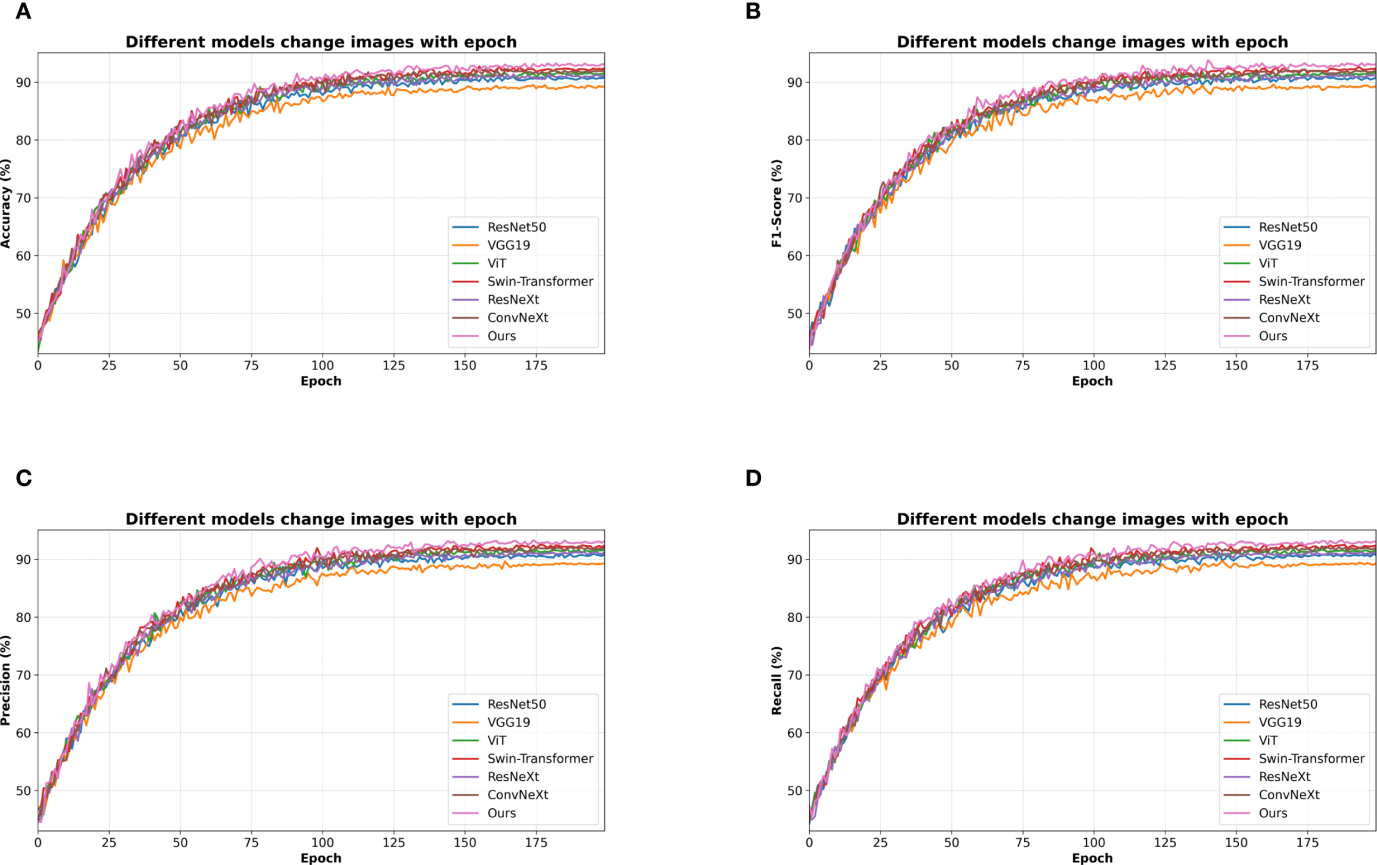
Training performance curves of different models. Each subfigure shows one evaluation metric across training epochs. **(A)** Accuracy of different models over Epochs **(B)** F1-Score of different models over Epochs **(C)** Precision of different models over Epochs **(D)** Recall of different models over Epochs.

This paper also gives images of how the evaluation indicators of different algorithms change with epoch on a self-made dataset, and the experimental results are shown in **Figure 6**.

### 5.2 Ablation experiment results

To further validate the effectiveness of the Bidirectional Transformer and Gradient Optimization Module in enhancing model performance, we conduct a series of ablation experiments on both the TCMP-300 dataset and the self-constructed Chinese herbal image dataset. All experiments are based on the Swin Transformer Base architecture, and we systematically remove each of the proposed modules to perform controlled comparisons. The complete ablation results are summarized in **Table 5**.

**Table 5:** Ablation study on the TCMP-300 and self-constructed datasets.

Method	Accuracy (%)	Precision (%)	Recall (%)	F1-score (%)
Self-Constructed Herbal Dataset					
Baseline (Swin-Base)	91.57	91.26	90.83	91.04
+ Bidirectional Transformer	92.13	91.80	91.43	91.61
+ Gradient Optimization Module	92.21	91.94	91.57	91.75
Ours (Full Model)	92.75	92.41	91.94	92.17
TCMP-300 Dataset					
Baseline (Swin-Base)	89.21	88.92	88.15	88.53
+ Bidirectional Transformer	89.74	89.43	88.72	89.07
+ Gradient Optimization Module	89.85	89.60	88.91	89.25
Ours (Full Model)	90.32	90.01	89.43	89.72

Across both datasets, the baseline model demonstrates a certain degree of global modeling capability; however, it lacks mechanisms for semantic direction modeling and gradient-guided enhancement, which limits its ability to represent complex texture structures effectively. The incorporation of the Bidirectional Transformer significantly improves the model’s capacity to capture contextual dependencies. Further integrating the Gradient Optimization Module provides a more substantial performance gain, as it directly enhances the discriminative power and stability of channel responses by leveraging gradient feedback. This mechanism enables stronger semantic coupling and improved generalization performance, which explains why its contribution is particularly prominent in the ablation results.

### 5.3 Grad-CAM visualization results on the custom dataset

**Figure 7:**
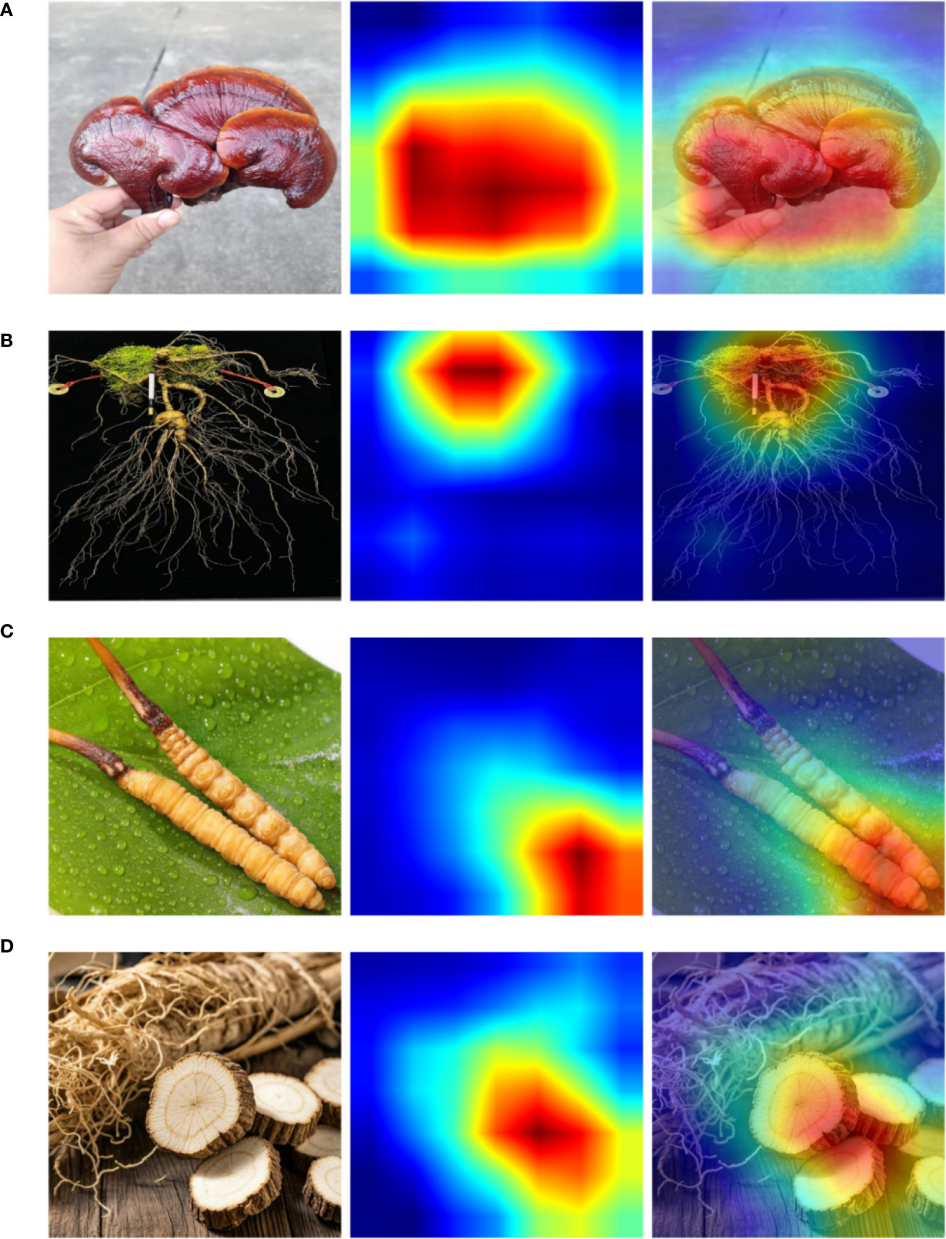
**(A–D)** Grad-CAM visualizations of representative samples from the custom herbal dataset.

To further analyze the model’s attention mechanisms over key regions, we employ Gradient-weighted Class Activation Mapping (Grad-CAM) to visualize representative samples from the self-constructed Chinese herbal dataset. Grad-CAM combines backpropagated gradients with channel-wise feature activations to generate weighted localization maps, effectively revealing the discriminative regions that contribute to the network’s decision-making process. These visualizations provide spatial insights into the model’s classification logic, allowing us to evaluate its attention distribution and feature focusing ability across different categories. Such analysis offers intuitive evidence for understanding the model’s interpretability and robustness. The experimental results are shown in **Figure 7**.

From the visualization results, it is evident that the model is capable of accurately focusing on key structural regions within herbal images during the recognition process. For instance, in the sample of *Ganoderma lucidum* shown in **Figure 7A**, the high-response regions highlighted by Grad-CAM closely align with the main contour of the mushroom cap, indicating that the model effectively captures representative local texture features during classification. Similarly, in the example of *Cordyceps sinensis* in **Figure 7C**, the attention is concentrated around the junction between the insect body and the fungal stem—an area that also holds strong discriminative significance in manual identification—further confirming the rationality and consistency of the model’s decision-making basis.

Moreover, **Figures 7B, D** demonstrate that even in the presence of complex backgrounds or partial occlusions, the model exhibits strong robustness by accurately localizing critical discriminative regions such as rootstocks or floral structures. This robustness can be largely attributed to the incorporation of the Bidirectional Transformer and Gradient Optimization Module, which respectively enhance the model’s semantic alignment and generalization capacity from the perspectives of contextual modeling and gradient-aware feature refinement. Therefore, the proposed model not only achieves superior performance in quantitative metrics but also demonstrates strong structural awareness and interpretability through qualitative analysis.

### 5.4 t-SNE visualization results on the custom dataset

**Figure 8:**
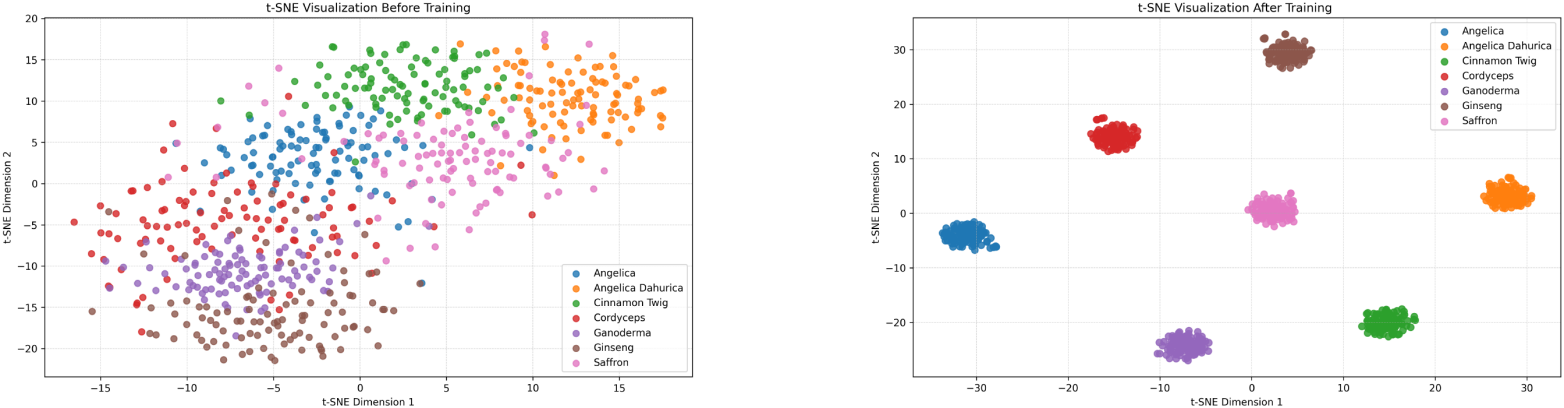
t-SNE visualizations of feature distributions before (left) and after (right) training on the custom herbal dataset.

To further validate the model’s discriminative capability in the feature space and its effectiveness in inter-class boundary separation, we employ t-distributed Stochastic Neighbor Embedding (t-SNE) to visualize the learned feature embeddings. Specifically, we extract intermediate layer outputs from the model both before and after training as the projection input, and compress the high-dimensional feature representations into a two dimensional plane. This allows us to observe the clustering distribution of features at different training stages and intuitively illustrate the evolution of the model’s feature separation ability throughout the learning process (**Figure 8**).

As illustrated in the visualization, the feature space prior to training exhibits significant overlap and intermixing among different classes, with no clear boundaries, indicating that the model in its initial state lacks effective discriminative capability. In contrast, after training, the feature embeddings demonstrate enhanced clustering behavior, with distinct and well-separated clusters emerging for each category in the low-dimensional space. This transformation highlights the model’s ability to effectively capture intra-class consistency and inter-class separability during the learning process. The structurally optimized distribution of features provides strong evidence that the proposed method achieves robust generalization and discriminative power in both feature extraction and semantic representation.

### 5.5 Feature map visualization results on the custom dataset

**Figure 9:**
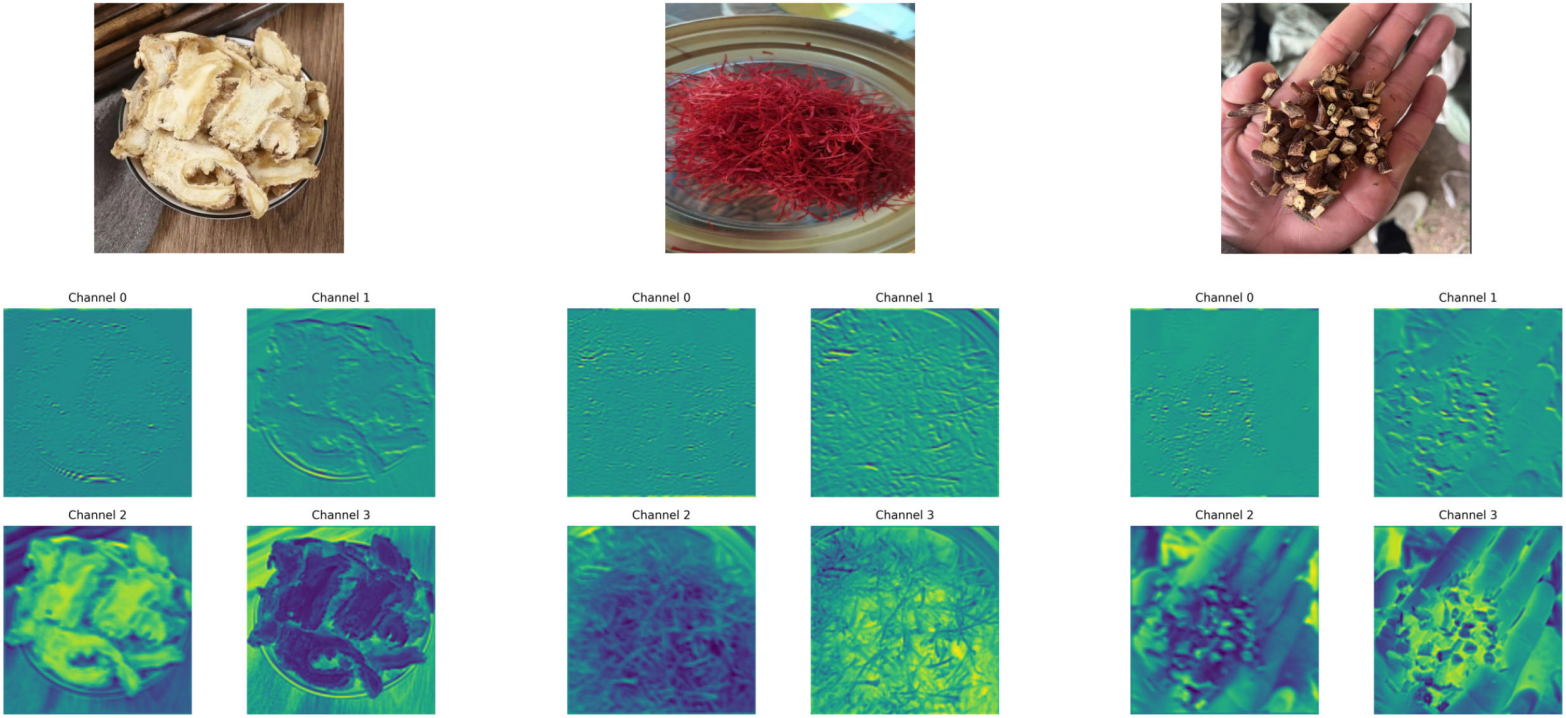
Feature map visualizations of different layers on representative samples from the custom herbal dataset.

In deep neural networks, visualizing feature maps across different layers provides valuable insights into how the model perceives and responds to input images along the spatial dimension. This process helps elucidate the underlying attention mechanisms and decision-making cues. To this end, we extract activation maps from intermediate layers of the proposed model on the self-constructed herbal image dataset and visualize the response regions across different semantic levels. This allows us to explore the network’s progressive enhancement in channel selection and spatial representation, thereby deepening our understanding of its hierarchical feature modeling capabilities (**Figure 9**).

As observed from the visualization, the model’s shallow-layer channels primarily focus on texture edges and local structural details of the input images. These low-level semantic features are consistently highlighted across various herbal categories, often manifesting as strong activations along contour lines, texture flows, and other fundamental visual elements. As the network depth increases, the mid- to high-level channels progressively attend to semantically meaningful regions, such as the morphological contours, tissue structures, and even central discriminative parts of the herbs. This indicates that the model successfully performs hierarchical abstraction from low-level perception to high-level semantic representation.

Moreover, the feature maps in the higher layers exhibit class-specific activation patterns, suggesting strong inter-class separability. The stability and selectivity of this spatial response behavior reflect the enhanced representational capacity achieved through the synergy between the channel attention and gradient optimization mechanisms. These findings further support the effectiveness of the proposed architecture in fine-grained image recognition tasks, particularly in terms of its discriminative strength and semantic understanding.

### 5.6 Visual analysis of difficult categories

**Figure 10:**
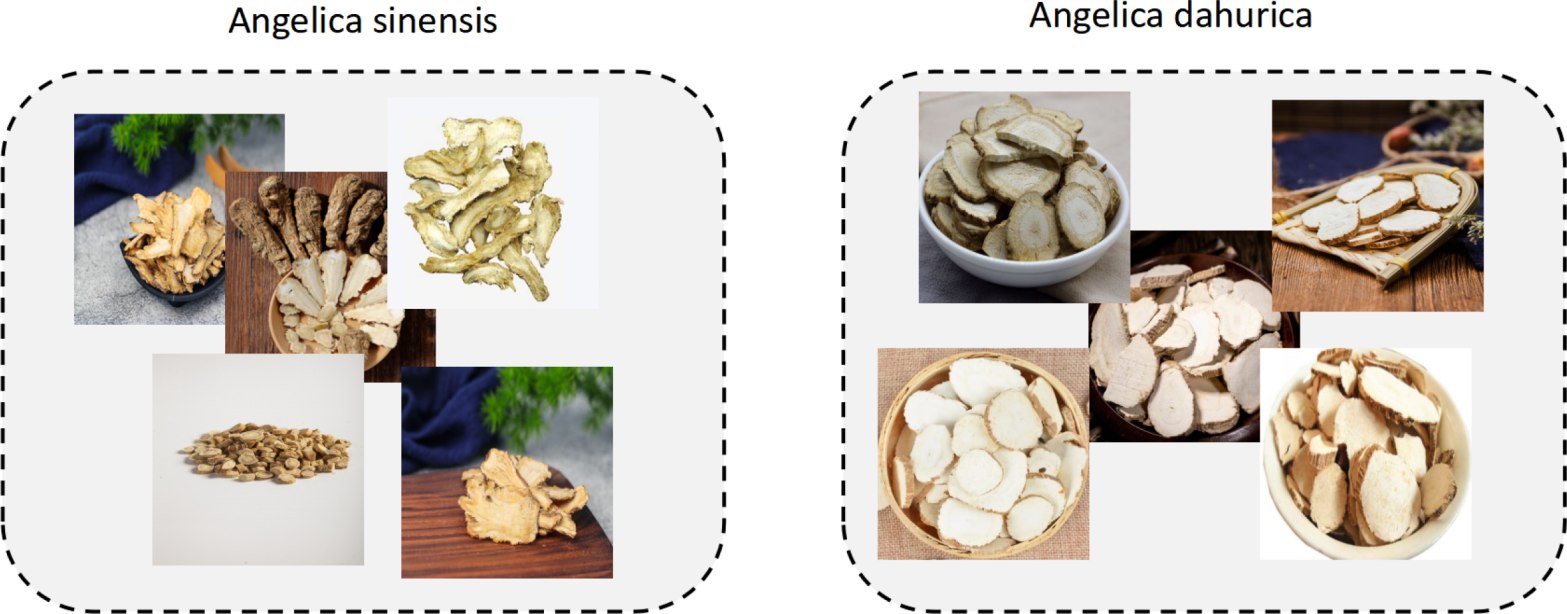
A direct comparison of Angelica sinensis and Angelica dahurica.

After conducting a comprehensive evaluation of the entire recognition framework, this study further performs a fine-grained visual analysis focusing on difficult categories to verify the model’s discriminative advantages in fine-level differentiation. We selected two commonly confused medicinal herbs in real-world applications—Angelica sinensis and Angelica dahurica—for comparison, as illustrated in **Figure 10**.

**Figure 11:**
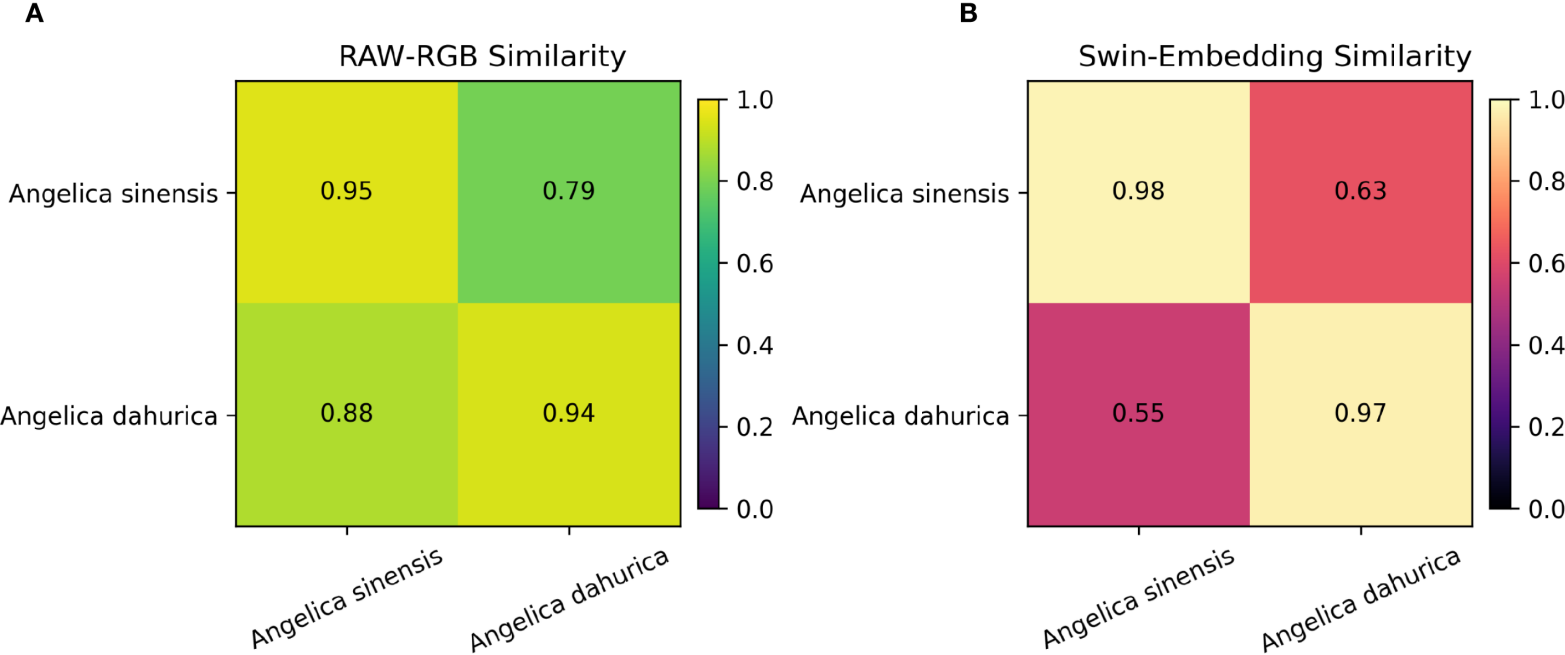
Comparison of cosine similarities between Angelica sinensis and Angelica dahurica in **(A)** raw RGB space and **(B)** Swin Transformer embedding space, demonstrating improved class separability with deep features.

As shown in the figure, Angelica sinensis and Angelica dahurica are highly visually similar in the original RGB images. To quantitatively assess this phenomenon, we first compute the mean RGB pixel vectors for each category after scale normalization and calculate the cosine similarity between the corresponding category centroids. Subsequently, a 1024-dimensional deep semantic embedding is extracted from each image using the proposed model. The centroid embedding for each category is then obtained, and the cosine similarity is recomputed in the embedding space. The experimental results are visualized as a pair of heatmaps, as illustrated in **Figure 11**, clearly demonstrating the enhanced discriminative ability of the model for visually confusing categories in the semantic feature space.

The experimental results show that in the original RGB space, the inter-category similarity between Angelica sinensis and Angelica dahurica is relatively high (up to 0.88), indicating that there is obvious confusion between the two at the visual level; while in the deep feature space extracted by the algorithm in this paper, the inter-category similarity drops significantly to 0.55, and the intra-class similarity of each is maintained at a high level (0.97-0.98), indicating that the model can effectively capture more discriminative semantic features, thereby enhancing the ability to distinguish easily confused categories, verifying its advantages in fine-grained recognition tasks.

### 5.7 Model interpretability analysis

**Figure 12:**
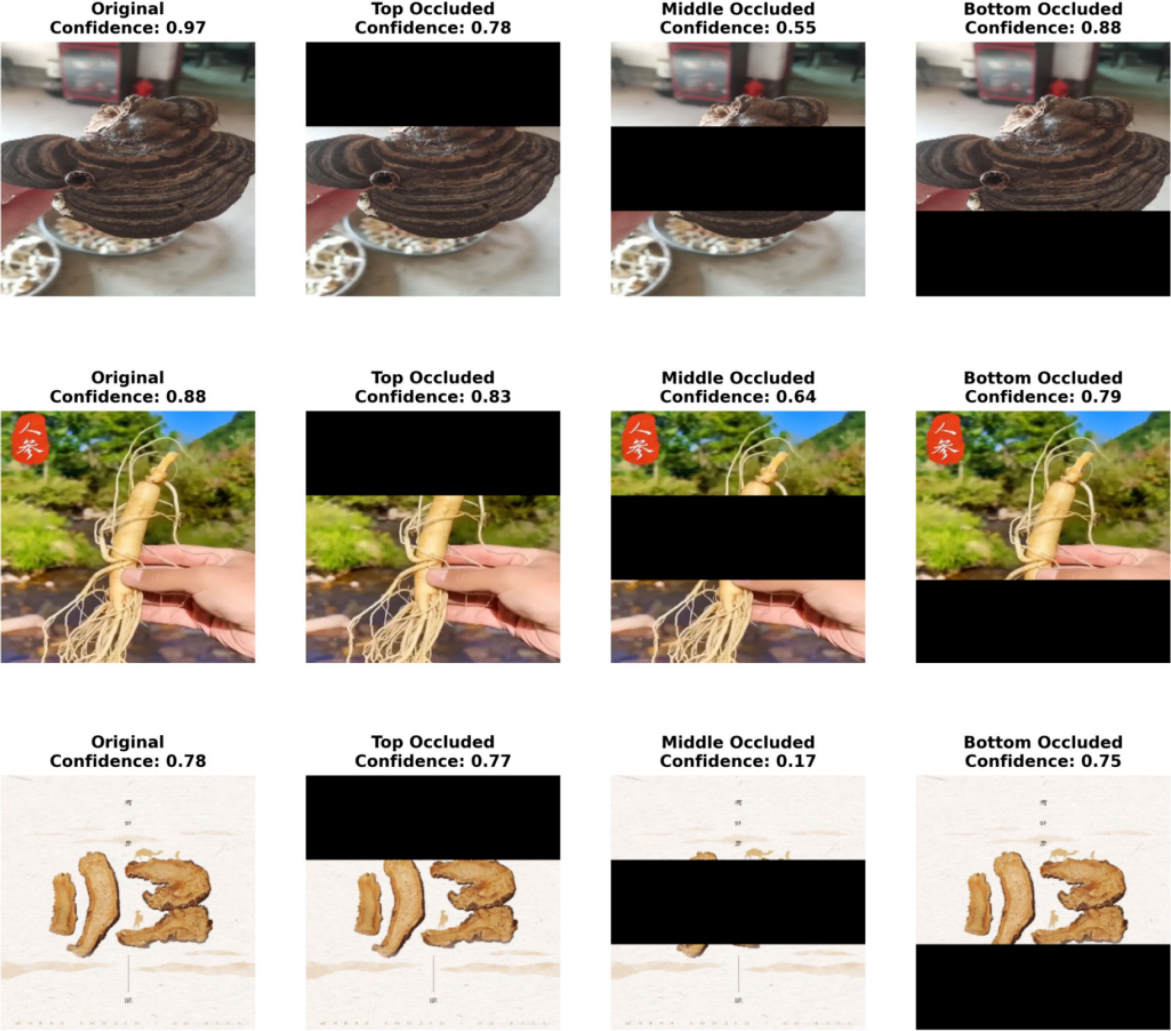
Results of the counterfactual interpretability experiment. The original images of three traditional Chinese medicinal materials (Ganoderma lucidum, Ginseng, and Angelica sinensis) and the changes in prediction confidence under different occlusion positions (top, middle, and bottom) are shown to verify the discriminant stability and semantic dependency of the model under the condition of missing key structures.

To further validate the reliability of the model in terms of structural awareness and discriminative reasoning, this study introduces a counterfactual explanation experiment to analyze structural perturbations in herbal images. Specifically, we simulate the absence of critical and non-critical regions by occluding the upper, middle, and lower parts of the original images, respectively. By comparing the model’s confidence scores under different occlusion conditions with those of the original images, we can intuitively reveal the model’s dependence on semantic structures and the stability of its decision-making logic. The experimental results are illustrated in **Figure 12**.

As shown in **Figure 12**, occlusions at different spatial locations have markedly distinct impacts on the model’s prediction confidence, highlighting its sensitivity to the structural regions of herbal images. Taking Ganoderma lucidum as an example, occlusion in the middle region causes a sharp drop in confidence from 0.97 to 0.55, indicating that the model heavily relies on the transitional area between the cap and the base during recognition. In contrast, occlusion at the bottom has minimal effect, suggesting that the root region contributes little to classification. Similarly, Panax ginseng also exhibits a notable confidence drop when the middle region is occluded, reflecting the model’s sensitivity to the junction between the main root and fibrous roots.

The case of Angelica sinensis further reinforces the model’s dependence on central semantic structures: occluding the middle region leads to the most dramatic confidence decrease, from 0.78 to 0.17, indicating that the model’s decision-making is primarily based on the central axis rather than peripheral slices. Overall, this experiment confirms that the model possesses structural awareness, focusing on the most discriminative core regions within herbal images for classification, while maintaining robustness against perturbations in non-critical areas.

### 5.8 Cross-dataset validation

In order to further assess the robustness and generalization ability of our proposed framework, we conduct cross-dataset validation experiments across both the large-scale TCMP-300 benchmark and our self-constructed herbal dataset. The experimental results are shown in **Table 6**.

**Table 6:** Cross-dataset validation results: models trained on TCMP-300 and tested on the self-constructed dataset.

Model	Accuracy (%)	Precision (%)	Recall (%)	F1-score (%)
ResNet50	64.25	63.81	63.47	63.64
ResNeXt	66.02	65.54	65.13	65.33
ConvNeXt	67.18	66.79	66.41	66.60
ViT	62.47	61.92	61.55	61.73
Ours	70.36	69.88	69.41	69.64

In cross-dataset validation experiments, we observed that the accuracy of all models decreased when migrating from the TCMP-300 dataset to our own dataset. However, our proposed method maintained state-of-the-art performance, achieving an accuracy of 70.36%, significantly exceeding mainstream baseline models such as ResNet50, ResNeXt, ConvNeXt, and ViT. These results demonstrate that despite inter domain differences and shifts in sample distribution, the proposed framework, leveraging the structure-aware capabilities of the bidirectional Transformer and gradient optimization modules, is able to better capture the discriminative features of medicinal material images, demonstrating enhanced robustness and generalization.

## 6 Discussion

### 6.1 Discussion of experimental results

Although the proposed framework achieves an improvement of approximately 1.1% over the Swin-Base baseline on the TCMP-300 dataset, we note that such a margin is non-trivial in the context of fine-grained herbal image recognition. Due to the high inter-class similarity and subtle structural variations among herbal categories, even small gains often reflect a substantial enhancement in feature discriminability and robustness. Furthermore, this improvement is consistent across multiple evaluation metrics and datasets, suggesting that the gain is not incidental but rather stems from the proposed structural modeling and gradient-guided optimization mechanisms. Therefore, while the absolute numerical increment may appear modest, its practical significance lies in providing more reliable and interpretable recognition performance in challenging real-world scenarios.

### 6.2 Qualitative analysis of visualization results

While visualization techniques such as Grad-CAM and t-SNE are inherently qualitative, they remain valuable for providing intuitive insights into the model’s decision-making process. These methods do not yield quantitative guarantees but effectively reveal whether the network attends to discriminative herbal structures such as veins and rhizomes. Their consistency with classification outcomes across datasets further supports the validity of our framework’s structural awareness and interpretability.

## 7 Conclusion

In this work, we proposed a structure-aware deep learning framework for fine-grained Chinese herbal image recognition. The framework is built upon a Swin-Transformer backbone and integrates a Bidirectional Transformer module with a Gradient Optimization Module. By incorporating graph-inspired structural modeling and gradient-guided channel selection, the model effectively enhances semantic discriminability and captures multi-scale dependencies, addressing the challenges of structural complexity and weak inter-class separability.

Extensive experiments on the large-scale TCMP-300 dataset and a self-constructed herbal dataset demonstrate that our framework consistently outperforms mainstream recognition approaches, including CNN with attention, cross-modal fusion, and lightweight transformers. Beyond superior quantitative results in accuracy, precision, recall, and F1-score, visualization analyses further confirm the framework’s structural awareness and semantic interpretability. These contributions highlight the advancement of our method over prior models and its potential to serve as a robust and interpretable solution for herbal recognition in sensor-based intelligent systems.

In future work, we intend to explore the integration of few-shot learning and cross-modal fusion techniques to further improve the adaptability and robustness of the model in data-scarce environments. Additionally, combining herbal image recognition with other TCM diagnostic modalities—such as tongue diagnosis and pulse signal analysis—holds promise for constructing intelligent and clinically applicable TCM-aided systems. Finally, we will focus on lightweight model design and interpretability improvements to facilitate deployment on mobile and edge platforms, accelerating real-world application and clinical adoption.

## Data Availability

The TCMP-300 dataset analyzed in this study is publicly available at https://figshare.com/articles/dataset/TCMP-521 300/29432726. The self-constructed herbal dataset is not publicly available due to institutional restrictions but can be obtained from the corresponding author upon reasonable request.
